# Knowledge, Attitudes, and Practices Concerning Malaria in Pregnancy: Results from a Qualitative Study in Madang, Papua New Guinea

**DOI:** 10.1371/journal.pone.0119077

**Published:** 2015-04-20

**Authors:** Erin V. W. Andrew, Christopher Pell, Angeline Angwin, Alma Auwun, Job Daniels, Ivo Mueller, Suparat Phuanukoonnon, Robert Pool

**Affiliations:** 1 Barcelona Centre for International Health Research (CRESIB, Hospital Clínic—Universitat de Barcelona), Barcelona, Spain; 2 Bridge HIV, San Francisco Department of Public Health, San Francisco, California, United States of America; 3 Centre for Social Science and Global Health, University of Amsterdam, Amsterdam, The Netherlands; 4 Papua New Guinea Institute of Medical Research, Madang, MP 511, Papua New Guinea; 5 Infection & Immunity Division, Walter & Eliza Hall Institute, 1G Royal Parade Parkville, VIC, Australia; Ehime University, JAPAN

## Abstract

**Background:**

Malaria is the leading cause of illness and death in Papua New Guinea (PNG). Infection during pregnancy with *falciparum* or *vivax* malaria, as occurs in PNG, has health implications for mother and child, causing complications such as maternal anemia, low birth weight and miscarriage. This article explores knowledge, attitudes and practices concerning malaria during pregnancy and it’s prevention in Madang, PNG, a high prevalence area.

**Methods:**

As part of a qualitative study in Madang, exploring MiP, participatory techniques (free-listing and sorting) were conducted along with focus group discussions, in-depth interviews (with pregnant women, health staff and other community members) and observations in the local community and health facilities.

**Results:**

The main themes explored were attitudes towards and knowledge of MiP, its risks, and prevention. Although there was a general awareness of the term “malaria”, it was often conflated with general sickness or with pregnancy-related symptoms. Moreover, many preventive methods for MiP were related to practices of general healthy living. Indeed, varied messages from health staff about the risks of MiP were observed. In addition to ideas about the seriousness and risk of MiP, other factors influenced the uptake of interventions: availability and perceived comfort of sleeping under insecticide-treated mosquito nets were important determinants of usage, and women’s heavy workload influenced Chloroquine adherence.

**Conclusion:**

The non-specific symptoms of MiP and its resultant conflation with symptoms of pregnancy that are perceived as normal have implications for MiP prevention and control. However, in Madang, PNG, this was compounded by the inadequacy of health staff’s message about MiP.

## Background

Worldwide, over 125 million pregnancies occurred in areas of malaria transmission in 2007[1] and malaria accounts for an estimated 10,000 maternal and 200,000 neonatal deaths per year.[2] Pregnant women are more susceptible than non-pregnant women to malaria[3] due to the immunosuppression that accompanies pregnancy (and this also might be related to increased levels of cortisol and estrogen). Furthermore, malaria infected erythrocytes can easily accumulate in the placenta. Antibodies directed against the surfaces of infected erythrocytes in the placenta are important in protection, and are usually absent in first pregnancy.[4] Pregnancy-related immunosuppression also increases the chance of severe malaria and related mortality compared to other adults.[3] Infection during pregnancy with any *Plasmodium* species can be harmful to mother and child, causing complications, such as maternal anemia and reduced birth weight caused by preterm delivery and fetal growth restriction as well as miscarriage and still birth, most prominently in first or second pregnancies.[3, 5–9]

In Papua New Guinea (PNG), malaria is the leading cause of illness and death, and the second most common cause of admission to hospital.[10, 11] The burden of malaria infection and related morbidity is highest amongst pregnant women and young children,[12–16] and while there are no exact estimates of the burden of malaria during pregnancy (MiP), birth weight patterns suggest that malaria is one of the major causative factors of low birth weight in lowland and coastal parts of the country.[17] Comparisons between malaria-endemic and malaria-free regions indicate that MiP accounts for up to 11% of anemia and 40% of low birth weight in the malaria-endemic, coastal areas.[17] Moreover, MiP is the leading cause of outpatient attendances, the third most common cause of hospital admissions, and the second commonest cause of death among pregnant women.[17]

As a result of the heterogeneous transmission settings, coexistence of multidrug-resistant *P*. *falciparum* and *P*. *vivax* parasites, and different vectors, the consequences of MiP in PNG are unique. Women may have little or no immunity to a particular malaria species, further increasing the possibility that an infection is fatal to mother or child.[18] Women in their first pregnancy are at greater risk of suffering from malaria-related morbidity and mortality. Data from PNG have shown that delivery infection rates were higher in primigravidae (up to 40%) and the average prevalence of infections at any antenatal care (ANC) visit was 34% in primigravidae, 30% in secundigravidae, 19% in multigravidae.[17] Although the impact on birth weight of first-borns is similar to those in sub-Saharan Africa, the effects of symptomatic malaria in pregnancy are more prominent in the Asia-Pacific region in general (maternal death, miscarriage, still birth, or premature labour).[18]

In PNG, before 2003, investment in malaria control was limited. However, a $147million grant (the highest outside of Africa and approximately $25 per person at risk of malaria) from the Global Fund changed this. From 2009 to 2014, over six million insecticide treated nets (ITNs) will therefore be distributed free of charge.[19] By comparison, about one million long-lasting insecticidal nets (LLINs) were distributed between 2006 and 2008 and a 2006 Demographic and Health Survey estimated that 33% of households owned at least one ITN.[20] A 2007 study of 1392 households indicated that 67% owned LLINs and that 40% of 1135 children slept under one the previous night.[21]

Current national MiP prevention policy prescribes a treatment course of first-line anti-malarial treatment, chloroquine and sulphadoxine/pyrimethamine (SP) at the first formal ANC visit, followed by weekly chloroquine prophylaxsis and iron and folate supplementation. Although rates of placental malaria infection are likely to have decreased in areas where LLIN coverage and the distribution of anti-malarials to pregnant women has increased[17], the extent of this impact is yet to be quantified.[22]

Although considerable qualitative research on the factors affecting uptake of interventions for malaria in pregnancy in sub-Saharan Africa has been conducted,[23] no such literature exists for PNG.[18] In general, social science literature on malaria in PNG is scarce and examines issues such as reasons for non-use of mosquito nets,[19] uptake of malaria treatment,[24] and community response to intermittent preventive treatment of malaria in infants (IPTi).[25]

In light of the limited qualitative research in PNG concerning MiP, the unique consequences of MiP in PNG, and considering that context-specific, socio-cultural factors must be taken into account for MiP interventions to be effective, [7] [17] [26] this article aims to describe two key areas of MiP prevention in Magang Province, PNG: local concepts of malaria and risk perception during pregnancy; and attitudes and behaviors related to malaria prevention during pregnancy.

## Setting and Study Population

Situated in the southwestern Pacific Ocean, PNG has an extremely diverse population of 6.5 million: 850 indigenous languages and thousands of disparate communities. Currently healthcare is provided at Aid Posts, Health Sub-Centers, Health Centers, District Hospitals and Provincial Public Hospitals, in ascending order of size and resources. Although some are government facilities, approximately 45% (and 49% in rural areas) are church-run.[27]

Data were collected in the Sumkar and Madang Districts, in the coastal Madang Province (population: 487,460; area; 28, 886 km^2^) from February 2010 to January 2011. Local industries in the area include tuna processing, engineering and joinery workshops, timber milling, and the manufacturing of black twist tobacco. In 2008, in Madang Province there were two doctors at the main hospital (Modilon), 20 Health Extension Officers, 106 nursing officers, 191 Community Health Workers at health centers and 124 staff at aid posts.[11]

Although the majority of MiP cases in Madang are *Plasmodium falciparum*, there are also cases of *Plasmodium vivax*.[28,29] In high endemic areas of PNG, such as Madang, acquired immunity to malaria can be common, thus malaria-related maternal mortality less likely though other effects of MiP remain. In such transmission contexts, MiP is often asymptomatic and detection less likely.[30]

This study was focused around three areas, each surrounding a health facility: Modilon Hospital in Madang town, Yagum Health Center, and Mugil Health Center on the north coast highway. The main language groups in the Yagum and Mugil areas are Amele and Bargam, respectively. In Madang town, participants came from many different language groups due to the high number of migrants. Modilon is government-run, whereas Yagum and Mugil are part of Lutheran and Catholic missions, respectively These facilities were selected for their proximities to Madang town: Modilon in Madang town, Yagum clinic a few kilometers out of town and the site of the PNG Institute for Medical Research (IMR), and Mugil clinic about 60 kilometers along the North Coast road out of Madang.

## Methods

### Study Procedures

The data collection was conducted by a Barcelona-based social scientist in collaboration with fieldworkers, fluent in the local language and with social science research experience. Data collection methods included free-listing and sorting of terms and definitions with individuals focus group discussions, in-depth interviews, observations in health care facilities and case studies of pregnant women (Table 1).

**Table 1:** Study Respondents and Data Collection Tools.

	Data Collection tool			
Type of respondent	Free listing and sorting	IDI	FGD	Case studies
Community members (women)	8		30	
Community members (men)	5		14	
Pregnant woman (PW)	16	52		27
Women with Babies (WB)		7		
Relatives of pregnant women		16		
Community Leaders	1	12		
Health Care Providers (HCP	7	7		
**Total**	**45**	**94**	**44**	**27**

Direct informal observations were carried out in the waiting areas and in examination rooms during ANC clinic hours (by EA, AA and AA) at the three health care facilities on multiple occasions during the initial phase of data collection to gather contextual data and observe patient-healthcare provider interactions, including waiting room educational talks, clinical examination and pill dispensing. Observations were recorded in the daily field notes that all three data collectors updated throughout the study. During the first weeks of data collection, free-listing and sorting was conducted with 37 participants—including pregnant women (16), health care providers (7), and general community members (14)—in a variety of settings (clinic waiting areas, before in-depth interviews, and during village visits). Free listing comprised of asking participants to list the top problems in pregnancy that came to mind. Participants explained each term and ranked it by seriousness and commonness. Participants responded in tok pisin, English or both depending on their preference. These terms were entered into excel ranking sheet in alphabetical order and frequency of terms and their rating were counted. These data were used to describe perceived commonness of different problems and to compile a list of terms used to describe common problems.

Nine focus group discussions (FGD) were held with groups of women or men in the communities, ranging from three to ten participants. A total of 94 in-depth interviews lasting from 45 minutes to 90 minutes were conducted: 52 with pregnant women, seven with women who had infants under one year of age (women with babies) (Table 2), 16 with relatives of pregnant women including husbands, parents, and siblings, 12 with community leaders, and seven with health care providers.

**Table 2:** Age and Parity of Pregnant Women and Women with Babies.

	Pregnant Women	Women with babies
**Age**		
≤ 20	10	0
21 to 30	26	2
30 to 39	14	4
≥ 40	2	1
**Number of living children**		
0 (1st preg)	10	0
1	14	1
2	9	1
3	10	1
4	3	0
5	3	1
≥ 6	3	3*

*one woman had 12 children

To gather more in-depth information throughout pregnancy and post-partum, 27 case studies were carried out. Case study women were visited on a monthly basis at their homes to discuss their pregnancies and experiences with and knowledge of ANC. Their husbands and other relatives were interviewed where possible.

The numbers of free-listing, interviews, and FGDs, were determined by the point of saturation (when no more novel information emerged).

### Participants and Recruitment

Participants were recruited through a combination of random, convenience and purposive sampling. Initially, during on-site visits at the three health care facilities all pregnant women present at antenatal clinic were invited to participate in an interview. Additional participants were recruited via a snowballing technique in various villages so as to reach pregnant women who may not have attended ANC or who otherwise would not have been captured in the initial phase. Finally, purposive sampling was used to ensure participants of varying ages, parity, marital status and gestational ages were interviewed. Convenience sampling was used to capture health care providers (HEOs and nurses) involved in ANC at the three healthcare facilities and community leaders identified in the surrounding villages (Tables 3 and 4). Case study women were selected based on gestational age (under 7 months), willingness to participate, and accessibility by the research team. An attempt was made to recruit an equal proportion of women from different age groups so as to ensure a diverse set of experiences with pregnancy (Table 4).

**Table 3:** Respondent characteristics.

	Opinion Leaders	HCPs	Relatives	PW	WB
**Area**					
Town	2	3	3	17	0
Yagum	4	3	5	16	5
Mugil	6	1	8	19	2
**Sex**					
Male	6	0	9	0	0
Female	6	7	4	52	7
**Type (OL)**					
Religious leader	4				
Community Leader	3				
Elder	3				
Other	2				
**Type (relative)**					
husband			9		
parent			5		
other			2		

**Table 4:** Case Study Characteristics.

Case Study number	Number months pregnant at initiation	Age	Married	Community	Number of living children	Relatives interviewed
**CS4**	4	24	Y	town	0	
**CS10**	6	24	Y	town	3	Husband
**CS12**	6	29	Y	town	2	
**CS23**	5	16	N	town	0	
**CS24**	7	19	Y	town	1	
**CS26**	6	19	Y	town	0	
**CS3**	5	27	Y	Yagum	1	husband
**CS7**	5	25	Y	Yagum	2	husband
**CS11**	6	21	N	Yagum	0	father
**CS15**	6	21	Y	Yagum	0	
**CS18**	6	24	Y	Yagum	1	mother
**CS19**	5	23	Y	Yagum	1	husband
**CS20**	7	17	N	Yagum	0	
**CS21**	6	40	Y	Yagum	6	
**CS22**	5	20	Y	Yagum	0	
**CS25**	7	42	Y	Yagum	4	
**CS27**	6	17	N	Yagum	0	
**CS1**	8	35	Y	Mugil	5	husband
**CS2**	5	28	Y	Mugil	3	husband, sister, mother, aunt
**CS5**	5	31	Y	Mugil	2	husband, sister, mother, aunt
**CS6**	6	42	Y	Mugil	8	husband
**CS8**	5	21	Y	Mugil	2	
**CS9**	5	27	Y	Mugil	4	
**CS13**	6	25	Y	Mugil	0	mother
**CS14**	6	20	Y	Mugil	1	
**CS16**	5	21	Y	Mugil	1	
**CS17**	5	27	Y	Mugil	1	mother

All interviews were carried out in English or Tok Pisin (the lingua franca in PNG) by (EA, AA, AA). In one case, a village elder who did not speak Tok Pisin was provided with a translator from her village.

Data collection was carried out using an iterative approach such that the research questions and interview question topics evolved as themes emerged from the data and as saturation was reached on a given subject area. Topics addressed included: problems during pregnancy; care seeking behavior during pregnancy; relationships with family members and healthcare providers; and knowledge or experience with malaria, specifically MiP. In-depth interviews began with a general discussion of problems during pregnancy and, if respondents did not explicitly mention malaria, the interviewer brought malaria into the discussion to ascertain their knowledge of malaria and the risks of MiP.

### Data Analysis

Group discussions and interviews were transcribed and translated into English if conducted in Tok Pisin. Free listing and sorting, observations and focus group discussions served to provide initial information regarding the topic area and target population. An initial codebook was developed using established categories based on the original research questions and was revised as new themes emerged from the data (through reoccurrence) and during research team meetings where the codebook was discussed and refined (EA and RP). The codebook was flexible and codes were reassessed during data collection and revised according to the emergence of novel themes. Likewise, as major trends and crosscutting themes emerged from the data, these were further investigated in the field.

Using the computer software Atlas.ti 6 (Scientific Software, Berlin, Germany), interviews and case study notes were coded and analyzed using the grounded theory approach whereby categories, themes and patterns emerge from the data.[31] In a second phase, data associated with the codes relevant to ANC attendance were extracted and organized by sub-themes so as to draw out key findings. A number of techniques were employed to improve reliability and achieve a comprehensive understanding of the findings: data from a variety of participants and different sources (focus groups, interviews and observations) were triangulated; three researchers carried out data collection to reduce individual bias; and case studies allowed for rapport-building and multiple interviews with that single respondent (not counted as separate interviews above) as well as verification of information in health booklets and through family member interviews.

## Ethical Considerations

This study was reviewed and approved by the Papua New Guinea Medical Research Advisory Committee (MRAC No. 09.01) and PNG Institute of Medical research Internal Review Board (IRB No.0905), the IRB of Hospital Clinic Barcelona and local procedures and requirements were followed. As approved by all ethics review committees and institutional review boards, and in accordance with the local ethics committee regulations, informed consent was obtained orally from study participants. Oral rather than written informed consent was obtained because the study procedures posed minimal risk to study participants and to avoid the possible negative influence of a written consent on rapport between researchers and respondents. With the agreement of participants, verbal consent was voice recorded prior to each interview or focus group discussion. Participation in the study was voluntary and a number of chances were given to participants to refuse interviews and they were informed of their right to not answer all the questions. If a participant agreed to be interviewed but appeared uncomfortable or unwilling to answer questions the interview was ended early.

## Results

The key themes of malaria and pregnancy that arose through this study were: knowledge; perceived cause; perceived symptoms; and prevention and treatment methods. There were no notable patterns of views across age, respondent type or clinic (Table 5).

**Table 5:** Problems in Pregnancy (free-listing).

Name of problem: vernacular term	Occurrences	English translation	Description
swollen/numb limbs; pains	74	abdominal pain; swollen legs; back ache; joints/legs numb	severe pain in the tummy; may lead to complication during birth; cannot sleep well; cannot walk around for long distances
Sot Blut; Ai raun	51	dizziness; lack of blood; anemia; pale skin	feels drowsy, tired and weak; lack of blood result in dizziness; shortage of blood may lead to complication during birth; feel lazy
Ino kaikai gut; traut; morning sik	33	loss of appetite; vomiting	loss of appetite
Fever; Malaria; Skin hot	30	fever; joint pains; vomiting; headache; loss of appetite; when lab test is done; hot skin	leads to/causes miscarriage; It can cause complication to woman when giving birth; leads to unconsciousness
Non-physical (Kros Planti; Wari)	11	depression and anxiety; worrying; nagging	worries during pregnancy leads to miscarriage or complication when giving birth; pregnant woman nags a lot and wants husband to help them; sometimes leads to quarrels and fights in the family
Sot Win; Kus; Kol Sik	6	asthma; cold sick; cough	cannot breathe well; cannot do work; coughs and feels weak; shivering
Skin Les	4	feeling lazy or dizzy	Does not want to do any work

### Knowledge of malaria and malaria in pregnancy

#### Terminology

Free listing of problems in pregnancy, with pregnant women, husbands, health care professionals and community leaders, revealed that the most common problems women reported were swollen limbs, numb limbs and body pains. Feeling dizzy or “lack of blood” were the second most common. The terms “malaria,” “fever” and “skin hot” were also listed as problems though not as frequently (Table 5).

During in-depth interviews, when discussing these problems experienced during pregnancy, some respondents spoke specifically of “malaria”, whereas others described malaria-like symptoms without using this term. Pregnant women and other community members used the words “malaria,” “flu,” “fever” or “skin hot” interchangeably and sometimes used these terms to describe sickness in general. The nurses interviewed used the word “malaria” when discussing the disease as well as the terms “skin hot” and “fever.”

#### Perceived Causes of Malaria

Across respondent groups, there were varied perceptions of the causes of malaria as well as those who reported not knowing the cause of the disease, pointing to overall community confusion (Table 6).

**Table 6:** Perceived causes of malaria.

Quotation	Participant	Theme
*If you don’t clean your area then mosquitoes will breed in empty tins—things like dog food plates*. *When lying there*, *they can collect water and mosquitoes can breed and bite us and we’ll have malaria*.	IDI, Case Study #26 female cousin, 23, Modilon	Perceived Causes of Malaria
*If there’s some conflict within [the family] and it hasn’t been talked about*, *it brings malaria*.	IDI, Pregnant woman, first pregnancy, 26, Yagum	Perceived Causes of Malaria
*Malaria occurs in pregnant mothers*. *When a pregnant mother faces problems or conflicts then it only results in malaria*.	IDI, Community Leader (male), Yagum area	Perceived Causes of Malaria
*Most women don’t take the of illness their baby or if they themselves get sick that serious; most women they say it’s due to problems at home that these illnesses occur*.	IDI, Nurse, Yagum clinic	Perceived Causes of Malaria

Among those who reported knowing a cause of malaria, there was a general sense that the disease is related to mosquitoes. For example, specific causes cited included not sleeping under a mosquito net, not taking malaria prophylaxis, or not using a mosquito coil. A few respondents said specifically that when a mosquito bites you it “leaves a parasite” in your body. This was the most in-depth knowledge of the mechanisms of infection explained, even amongst some health care providers.

Another perceived cause of malaria was lack of household hygiene as a general concept. Some did specify that this referred to keeping outdoor areas clear of thick grasses, garbage and containers that could potentially be mosquito breeding grounds. Personal hygiene factors, such as absence of hand-washing before eating or cooking or not having clean bedding, were also cited as causes of malaria.

Behavioral causes included drinking contaminated water, eating “bad” food, or having nutritional deficiencies (specifically lack of protein). Staying out in the cold for too long, carrying heavy loads, or coming into contact with “germs from flies” were also reported as causes of malaria. Many respondents explained that conflict and family disagreement caused illness in general and malaria specifically.

Malaria was sometimes described as a hereditary disease. Others understood that, once cured, it could not be contracted again or conversely as a chronic disease of which one is never cured.

When addressing MiP specifically, there was a general perception that pregnant women with malaria could transmit it to the baby. Malaria was also described as an expected part of pregnancy: “a normal sickness because I had a child” or a “sickness you get when you’re pregnant during the first and second months.”

#### Perception of Symptoms

In general, respondents found the symptoms of malaria difficult to identify, though the main symptoms of malaria were reported as “skin hot,” shivering, feeling cold on the inside, fever, dizziness, vomiting a yellow substance and loss of appetite. A number of women and health care workers mentioned having enlarged spleens or other spleen-related sickness. Others thought malaria could lead to seizures (Table 7).

**Table 7:** Perceived symptoms of malaria.

Quotation	Participant	Theme
*The sickness I have is in my spleen*. *If I stop taking the medicine my spleen will grow and when it reached mature stage it will make me sick*. *When I was expecting the baby I did not visit the clinic at an early stage*. *When I was four months pregnant my spleen expanded and it was rumbling against the baby and I couldn’t sit*. *I could hardly sit down or even sleep and after taking the medicine I felt ok*. *At times I feel the pains it comes from inside…I take Chloroquine for my spleen*.	IDI, Pregnant woman, 2 children, 31, Modilon	Perception of Symptoms
*When I sleep*, *my mouth and head will start aching—making me feel like it’s going to come now [like she’s going to give birth]*. *I would try to rise up only to find out that it’s too late because my legs and hands would be already stiff by then*. *And mum would start pulling my hair till I come back again*, *back to normal again*.	IDI, Pregnant woman, 2 children, 21, Mugil	Perception of Symptoms
*Ok the signs and symptoms of malaria come in different ways*. *One common way we normally see it is hot body*, *fever*. *Hot and cold body temperatures making a person shiver and wanting to be exposed to sunlight all the time*. *Plenty of people know about this symptom*. *Another symptom you may have is a stomachache*. *You will appear normal but still feel the stomachache*. *Also*, *headache and backache*, *even diarrhea*. *There are plenty signs and symptoms of malaria*. *Before we thought that there was only one and that was as I’ve mentioned; hot and cold body and shivering which I thought was the only sign of malaria*. *But now I am seeing that the symptoms and signs of malaria are many so it is quite confusing*. *I will appear normal—eating well and going about my business but because of stomachache I went to the hospital recently and they told me I tested positive for malaria*.	IDI, Community leader (male), 42, Yagum	Perception of Symptoms
*When my mother in-law gave birth to her last child she had a very big case of malaria*. *So when she delivered our little sister*, *she was born with her two ears blocked and right now they’re still blocked and she’s deaf*. *She told me the story herself and I am concerned and trying not to get malaria*. *That’s why I get scared*.	IDI, Pregnant woman, first pregnancy, 19, Modilon	Perception of Symptoms

**Fig 1:**
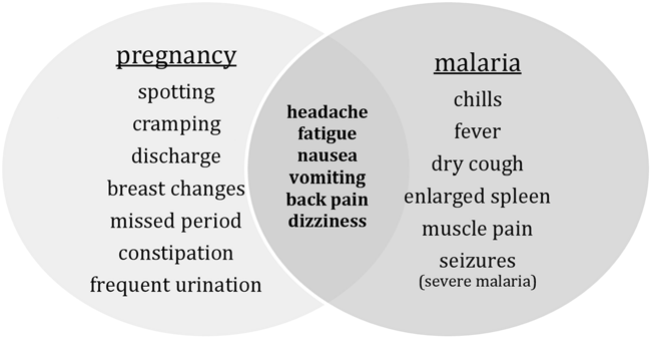
Potential Signs and Symptoms of Pregnancy and Malaria.

Because some of the signs of early pregnancy and the symptoms of malaria are occasionally the same, such as nausea, vomiting, fatigue and dizziness, women were not always able to differentiate between the two (Fig 1).

Babies affected by malaria while in the womb were perceived as likely to either be born with malaria and shows signs of the illness (such as a fever) a few weeks after birth, or to suffer mental impairments as a result. A few people reported deafness in the baby as a result of MiP.

### Malaria Prevention and Treatment

#### Knowledge of chloroquine prophylaxis

When available, chloroquine was provided as intermittent preventive treatment of malaria for pregnant women (IPTp) and as malaria prophylaxis. Women were instructed to take it every Sunday. It was common for respondents to refer to chloroquine by name and pregnant women who had attended ANC often knew it was a medicine that must be taken every Sunday. It was generally seen as protecting pregnant women and their babies against sickness. However, it was not necessarily understood to specifically protect against malaria (or “skin hot”), though some women did make this association (Table 8).

**Table 8:** Knowledge of Chloroquine prophylaxis.

Quotation	Participant	Theme
*No*, *they didn’t tell that and I didn’t know*. *The blood medicine is for providing us with more blood but chloroquine I don’t know*.	IDI, Case Study #14, 1 child, 20, Mugil	Knowledge of Chloroquine prophylaxis
*They said that is to prevent the malaria parasite from affecting the baby*. *For any other sicknesses we need to take our treatment*. *Any severe sickness could cause damage to the foetus*. *The chloroquine supplied was meant for the protection of malaria*.	IDI, Case Study #2, 3 children, 28, Mugil	Knowledge of Chloroquine prophylaxis
*If you do not take your malarial tablets*, *your blood will be full of parasites and of course your baby would also be affected*. *The baby will be born an unhealthy baby*. *That is the whole reason the medicine is supplied*.	IDI, Pregnant woman, 4 children, 23, Yagum	Knowledge of Chloroquine prophylaxis
*For chloroquine*, *I would take it with water but because it tasted very bitter I would shove it into ripe banana and gulp it down*.	IDI, Pregnant woman, 22, first pregnancy, Modilon area	Knowledge of Chloroquine prophylaxis

Observations along with reports from pregnant women and their husbands demonstrated that health care workers did not always inform women what the medicine was intended for and chloroquine could be confused with other medication, such as panadol, or viewed as preventing general sickness, such as fever and cough. Observations also highlighted a lack of explanations from health staff regarding the risks of MiP. Communication was largely one-directional and pregnant woman rarely asked questions.

In addition, messages from health care workers regarding pregnancy care were unclear. While chloroquine and iron tablets were provided at ANC visits, health staff told women to eat plenty of greens and protein-rich foods. Although health messages warned against anemia during pregnancy and loss of blood during delivery, they did not necessarily link this to iron supplementation, cholorquine, or malaria. Respondents who spoke about the importance of IPTp often mentioned the local research institution, the Institute for Medical Research (IMR) as having disseminated information and raised awareness about prevention of MiP.

#### IPTp Adherence

A number of factors affected whether women adhered to IPTp including perceived risk, support, side effects and availability of medication (Table 9).

**Table 9:** IPTp Adherence.

Quotation	Participant	Theme
*I have to take that early on because I don’t want my baby to get sick you see*. *And I’ve heard a lot of problems of babies born out of malaria mothers becoming very very sick and I said ‘oh*, *I don’t want my baby to get malaria so I must get chloroquine every Sunday’*. *So I take two tablets every Sunday*. *The earlier the better*.	IDI, Community Leader (female), 7 children, 55, Mugil	IPTp Adherence
*Like I said*, *it’s to prevent sickness*. *The most important thing is that I must bathe and stay clean for fear that if I am dirty any sickness will just easily come and attack me*. *So*, *I must stay clean or take whatever medicine is given to me so as to avoid getting sick*.	IDI, Pregnant woman, 4 children, 32, Mugil	IPTp Adherence
*Before taking this medicine I was just fine and able to do all kinds of things*. *However after taking it my legs and hands started to go numb*, *I felt skin cold*, *dizzy and want to sleep*. *I sometimes experience an upset tummy and feel like vomiting*. *I walk for a short distance and feel dizzy and want to fall down*. *This all happens when I take the medicine*. *So now I don’t want to take it*.	IDI, Case Study #3, 2 children, 22, Modilon	IPTp Adherence
*I took two tablets and then vomited one because of its bitter taste*. *I find it hard to swallow the medicine*. *I only take one and throw out the other tablet*.	IDI, Case Study # 2, 3 children, 28, Yagum	IPTp Adherence
*They say it is bitter*, *they say itchiness*. *Also sometimes they just forget*. *They do work work work and they forget to take their routine tablets*. *Work work work*. *That’s one of the main reasons*.	IDI, Sister in Charge, Mugil Health Center	IPTp Adherence
*They give me this chloroquine and I drink it only when I get sick*.	IDI, Mother of case study #5 and case study #2, Mugil area)	IPTp Adherence
*You know*, *I question the nurses like I am not sick why should I be taking chloroquine*?	IDI, case study #2, 2 children, 28, Mugil area	IPTp Adherence
*When I got sick while I was pregnant*, *I was afraid about the baby therefore I took medicine on that same day*. *But if I was not sick*, *I wouldn’t be bothered to take the medicine*.	IDI, Pregnant woman, 5 children, 35, Mugil area	IPTp Adherence
***I*: *So when you were pregnant*, *did you take chloroquine*?**	IDI, Nurse midwife,	IPTp
*R*: *Yes I did*, *but sometimes I forgot like when I was going to work I forgot to take it in the morning*. *For some maybe they don’t want to take it the chloroquine since it’s so bitter*. *People say that the coated one is ok but the other one is too bitter*.	Modilon hospital	Adherence

Understanding the risks of MiP, or fearing their baby would fall ill, encouraged women to take chloroquine every Sunday, even if it they preferred not to. Even if malaria was not seen as a serious illness during pregnancy, some saw taking chloroquine as part of the general routine of healthy living during pregnancy. Pregnant women were more likely to take chloroquine if encouraged by their family members. Those who had witnessed others suffer the consequences of malaria or who had a negative experience with MiP in a previous pregnancy were more likely to consider MiP as a risk and thus more likely to take chloroquine (Table 10).

**Table 10:** Perceived severity of malaria in pregnancy.

Quotation	Participant	Theme
*Malaria is one of the biggest problems in this village so now if the people are having little sickness like skin hot we conclude that they have malaria*.	IDI, husband of case study #1, Mugil	Perception of the Severity of Malaria
*Well because malaria is like common here and we start getting malaria like since we were*, *well we start getting malaria as soon as we’re born I guess*. *Or probably in childhood when we’re one month*, *two months*, *three months old do like for example*, *I myself have not missed malaria in a year*. *Every year I get like at least an episode of malaria*. *So because its quite a common thing*, *its not seen as very dangerous in pregnancy*. *A lot of women when they get sick they can’t get treatment you know in a relaxed manner and they go home again*, *yes so*, *until they have another complication and then when you say its due to malaria and they take it as something really dangerous*.	IDI, IPTp Study Doctor, Modilon	Perception of the Severity of Malaria
*Yes*, *there are plenty—plenty mothers who have malaria*, *when they are pregnant*, *this malaria kills the baby or sometimes it will kill the mother and she’ll die*.	IDI, Pregnant woman, 5 children, 35, Mugil	Perception of the Severity of Malaria
*There have been two mothers in the community who had a miscarriage*. *That’s when she (one of the mothers) was very severely sick with malaria*. *She got very very sick*. *We were going and visiting her but malaria conquered her and she sadly lost her baby*.	IDI, Community Leader (female), 51, Yagum	Perception of the Severity of Malaria
*I thought it would just be on the outside skin of my body*. *But when I got sick I realized that malaria was inside and caused me to get sick but I didn’t know that it would be in the womb*… *I thought it was just an ordinary sickness but after the two miscarriages I found out that malaria can destroy my body*.	IDI, Woman with baby, 2 children, 25, Mugil area	Perception of the Severity of Malaria
*We would all go together to the clinic*. *Sometime later she got very sick and was diagnosed with malaria—she was cold and shivering*. *The next day some mothers told me that this mother we use to come with for the clinic was dead due to malaria*. *So when I had malaria I was quick to get treatment and now I am fine*.	IDI, Woman with baby, 2 children, 25, Mugil	Perception of the Severity of Malaria

Non-adherence was linked to side effects and negative experiences associated with cholorquine, such as irritation or itchiness on the skin, dizziness, fainting, and vomiting. In addition, the bitter taste, or a dislike for medicine in general (not specifically during pregnancy) were also deterrents. There were also reports of tablets being too big to swallow. However, some women mentioned masking the bitter taste so as to make the medicine more palatable. Across all respondent groups it was also reported that women were too busy with housework, that they forget to take their medicine or that they were “lazy” (including self-reports).

Conceptions of chloroquine and malaria also affected adherence. Understanding chloroquine as curative rather than preventative deterred women from adhering to the prophylaxis and some pregnant women and their husbands explained that chloroquine should only be taken at the onset of symptoms and healthcare workers corroborated this as a common sentiment among their patients. This was the case even when people understood the dangers of malaria in pregnancy. Some women did understand that chloroquine could prevent malaria but used it as they saw necessary: for example, when they noted the presence of mosquitos. Perceptions of symptoms and causes of malaria—e.g. regarding malaria as hereditary; treatment results in immunity; or that it is an incurable chronic disease—also affected adherence.

Although most women in this study were able to attend ANC, unavailability of medication could prevent women from taking chloroquine. Some women reported that they were not given any chloroquine at the ANC or not the full dose. Health care professionals working at ANCs likewise complained about chloroquine stock outs.

#### Other Malaria Prevention Methods

Respondent reported other methods of malaria prevention (Table 11). Even those who did not cite mosquitoes as causing malaria reported that mosquito nets and mosquito coils were appropriate prevention methods. Since 2004, free LLINs have been distributed in villages throughout PNG and many of the respondents were recipients of these nets. The IMR was also often cited as a distributor of mosquito nets.

**Table 11:** Other prevention methods.

Quotation	Participant	Theme
*It’s not good for pregnant mothers to get malaria so they need to sleep under mosquito nets to protect themselves from malaria*.	IDI, husband of case study # 19, expecting first child, 28, Yagum area	Other prevention methods
**I: Are there some ways you can do to prevent malaria?**	IDI, Pregnant	Other
R: *Yes*. *Sometimes I get medicines from the hospital and other times I use bush medicines (herbs) like water that have been talked over it before I drink it*.	woman, 2 children, 24, Modilon	prevention methods

Although mosquito nets were observed in many houses, pregnant women did not always utilize them. Mothers reported putting infants or children under a net rather than sleep under it themselves, even during pregnancy. In addition to inadequate space, women reported that sleeping under a net was too hot or uncomfortable. Some women used nets for fishing and others reported that their nets were worn out or that supplies were limited at the clinic. Burning materials such as dried coconut shells to create smoke was another way to deter mosquitoes.

In addition to mosquito-specific prevention, some reported cleaning the house and beddings, clearing rubbish away, and maintaining personal hygiene as methods for preventing malaria. Dietary solutions such as eating greens (such as tulip and cabbage), papaya seeds, and protein were also described (and also for general pregnancy health). In addition, some reported included taking “bush medicines (herbs) that had been talked over before drinking” as an effective prevention method.

#### Knowledge of Malaria Treatment

Participants provided mixed reports of how to treat malaria. Both biomedical and non-biomedical treatment methods were cited, though these were not seen as mutually exclusive (Table 12).

**Table 12:** Malaria treatment.

Quotation	Participant	Theme
*If you know that you are sick or having malaria then you must go down to the hospital to get the medicine*. *When the adults have malaria it is ok but if the little kids have malaria we must quickly bring them to the hospital*.	IDI, case study #7, 3 children, 25, Yagum area	Malaria treatment
*If we feel cold and are shivering then we would already know that we have malaria*. *Then we will go to the hospital for the doctors to check us*. *If we have malaria then they will treat us with anti-malaria tablet*.	IDI, woman with baby, 1 child, 22, Yagum area	Malaria treatment
**I: Is malaria different if you are not pregnant versus pregnant?**	IDI, case study #1,	Malaria
R: *It’s the same sickness but when I got sick while I was pregnant*, *I was afraid about the baby so I took medicine on that same day*. *If I was normal*, *I wouldn’t be bothered to take it*.	5 children, 35, Mugil area	treatment
*In the past there was no medicine but people used the gorgor plant to wash the sick patient*. *This would make the fever will come down*.	IDI, Community Leader (female), Mugil area	Malaria treatment
**I: Do you use bush medicine for all the illnesses or for malaria only?**	IDI, Community Leader (male),	Malaria treatment
R: *The husk of the kwila tree is for malaria*, *coughs and knee problems*.	Yagum area	
*If I feel a cough or flu or something*, *I boil hot water and steam*, *eat citrus fruits*, *or use herbs from the village to stop the sicknesses*. *This makes sickness go away so I don’t get seriously ill very often*.	IDI, Case Study #6, 9 children, 42, Mugil area	Malaria treatment
*There is a leaf that grows from a plant near the river*, *that looks similar to a taro plant*. *You use the shoot to treat malaria*. *The shoot of the leaf is fried or cooked over the fire then eaten with powerful seeds*. *I was healed from it when I had a huge spleen while pregnant with my son*.	IDI, mother of case study #17, Yagum area	Malaria treatment
*Well apart from chloroquine*, *there are some other greens—vegetables that they take and they say that they feel better*. *It works like malaria tablets they say*. *Some kind of cabbages they grow in the gardens*, *fresh this cabbage from the bush*. *They say it’s like chloroquine*.	IDI, Sister in Charge, Mugil Health Center	Malaria treatment
*We steam ourselves and if it doesn’t help*, *we go straight to the hospital*	IDI, Community Leader (male), Yagum area	Malaria treatment
**I: When you have malaria do you do anything to stop the sickness?**	IDI, case study #7, 3 children,	Malaria treatment
R: *No*, *I depend solely on cold water*. *When I feel hot I wash with cold water but this skin hot will still be within me*. *They tell me to go down to the hospital but I refuse and just stay in the house*. *I just wait until the fever goes down*. *While staying home I always wash to cool myself*.	25, Yagum area	
*If someone is just sick the medicine is there to help cure her sickness*. *If she gets the right medicine for this particular sickness then she might be cured*. *But if someone is sick for a long time*, *then the village elders will call a meeting so that they can discuss her sickness and find out what really caused it*. *If they find the cause of the sickness then she will get better but if they talk and do not find the solution the medicine will not help her to get well*. *They can talk but this sometimes can lead to death*.	IDI, Community Leader (female), Mugil area	Malaria treatment
*In our custom*, *we have to gather together to sit down and straighten worries*. *She can talk about it or they can come together and be at peace- forgiving one another*. *As a mother*, *I must forgive and be in one piece with my daughters—they must forgive each other and resolve whatever wrong exists within in the family*. *They must reveal every single distress*.	IDI, Mother of case study #5 and case study #2, Mugil area	Malaria treatment
*I went and saw some church leaders and pastors and they did some counseling to me*. *I expressed the root or cause of my sickness or whatever I’ve said revealed myself and the sickness*. *They prayed over me so the sickness finished from me in 2008*.	IDI, Pregnant woman, 3 children, 39, Mugil area	Malaria treatment

Going to the hospital or clinic and taking medicine was seen as one option for treating malaria. Respondents mostly referred to “medicine” in general but sometimes specifically referred to “chloroquine.” There were reports of women being more likely to seek such biomedical treatment or take medicine if they were pregnant.

Non-biomedical care included symptom alleviation and treatment of malaria. Drinking, eating, steaming or washing with “gorgor” (a local ginger root plant), paw paw seeds or other “fragrant plants” were methods treat malaria and its symptoms.

Using non-biomedical remedies was often reported as a first line of treatment with hospital attendance occurring if these were not effective. In general there was a preference for home remedies over hospital attendance.

As many explained that the root causes of illness was related to disputes with family members or other community members, resolving these issues was often described as a key to treatment, even if done in conjunction with a visit to the clinic and taking of medicine. Praying to God or visiting a religious leader was also cited as method for curing malaria among those who were religious.

## Discussion

Knowledge, attitudes and practices surrounding MiP were inconsistent among all respondents at all three sites. While pregnant women and their relatives were concerned with the health of the fetus, opinions of how to best to achieve this were varied.

Similar to findings of multiple studies in sub-Saharan Africa,[7, 32–34] reportedly common side effects of pregnancy overlapped and were co-reported as symptoms of malaria, such as body aches, dizziness, vomiting, and shivers or fever. In some cases, malaria was described as a *result* of their pregnancy, as has been reported in Ghana, Kenya and Malawi.[35] In general, understandings about malaria and pregnancy in Madang were also melded with general ideas of health and disease. Although many participants identified mosquitoes as the cause of malaria, several other factors were cited, such as lack of hygiene and nutrition. Such perception of malaria causes as similar to other health issues has also been found in other settings.[35, 36] It is possible that, simultaneous messages around anemia, nutrition, and malaria communicated at ANC clinic, as well as the concurrent distribution of iron tablets and chloroquine, complicates perceptions of malaria and it’s prevention. In addition, although a particular clinical feature of malaria in PNG is high rates of enlarged spleens [12], women who reported having spleen-related sickness did not link it to malaria, which further suggests unclear messaging.

While access to ANC was not a salient barrier because most women in the study were able to attend, previous studies have shown that even when women attend ANC repeatedly, they may not have adequate and appropriate information about pregnancy.[37, 38] There was both reported and observed variability in health care workers’ explanations of the purpose of medicine provided. The observations presented above also revealed variability in the extent to which health care workers’ explained the risks of malaria during pregnancy, as has been found in other settings.[39, 40] For example, a study in Nepal found that little attention was paid to educating pregnant women about danger signs and possible complications. Furthermore, communication flowed mostly in one direction from health worker to pregnant woman,[40] as was the case in Madang.

Mosquito nets were not used when experienced as uncomfortable and were not always available. Previous qualitative research in PNG has shown that among a variety of human, environment and net-related factors (including heat, net lifespan, and mosquito density), indifference towards regular mosquito net use (or non-use), rooted in lack of fear of malaria is the most influential factor effecting why Papua New Guineans who own mosquito nets choose not to use them.[19] Nevertheless, while studies in other places have found fear of the negative impacts of insecticide-treated bed nets to be a key deterrent,[34] this was not the case in this study setting perhaps because of the more recent widespread availability has yet to result in the spreading of such fears.

A number of factors were linked to pregnant women’s uptake of and adherence to chloroquine. Many side effects of cholorquine were reported, including dizziness, vomiting and skin irritation. Other deterrents were the size of the pill and its bitter taste. Some reported that study drugs were sugar coated and tasted better than the medicine normally prescribed. Although sugar-coating chloroquine could possibly increase adherence, minimal research compared adherence over this variable[41] despite preliminary findings suggesting that sugar coating pills may help with the discomfort of taking them.[42]

Being busy with housework and forgetting to take medicine was another commonly reported reason for non-adherence to chloroquine, echoing the findings of a qualitative study that found that heavy workload is a key determinant of women’s health in PNG.[43] The opportunity cost of malaria prevention, including ANC attendance, is a neglected area of study.

Previous studies have found that there are three main categories of belief systems regarding illness in PNG: sickness caused by sorcery or witchcraft; sickness pertaining to the environmental or seasonal changes; and finally illness introduced by the 'white man', (such as cancer and diabetes).[44] As was found in the current study, people thus often pursue a combination of biomedical and traditional solutions to their health problems,[24] sometimes believing that biomedical interventions will only succeed once conflicts have been resolved. Similarly, as has previously been reported in PNG for health and illness in general, malaria and the associated pregnancy complications were sometimes seen as caused by family disputes or conflicts that can be cured with incantations over herbal drinks or betel nuts as well as conflict resolution.[45, 46]

Furthermore, there was a general understanding of biomedicine as curative rather than preventative, which deterred women from seeking IPTp and only attending clinic once they were sick. Other studies have shown this tendency to perceive biomedicine as curative rather than preventative.[47, 48] Prayer was also reported as a malaria treatment method as has been found in many settings for treatment and prevention of various diseases. For example, Christian prayer in South Africa was found to be essential for diabetes treatment.[49,50]

Lacking from these findings was mention of higher risk in adolescents or first pregnancies from any respondent.

## Policy Implications

While access to ANC was not an issue for the women in this study, it is nevertheless critical that the WHO recommendation for “focused” ANC be followed. This entails a minimum of four health facility visits (with the first occurring during the first trimester), if, following a standard risk assessment, the woman and her fetus are judged to be low risk.[51,52] Specifically, providing anti-malarials should be included in these visits and care should be taken to prevent shortages such as pre-ordering supplies including mosquito nets.

In addition, to improve understanding of MiP and it’s risks, healthcare providers could allow for increased two-way communication encouraging women to ask questions and perhaps checking their knowledge during ANC visits. Health education could focus on the causes, prevention and treatment of malaria as well as emphasizing the importance of ANC attendance even in the absence of illness. Finally, it is imperative that messages about the greater risk for MiP in adolescents and first-time pregnancies be disseminated.

Considering the multi-belief systems that exist surrounding illness and health, a multi-pronged approached which accounts for these is essential. The introduction of the PNG National Policy on Traditional Medicine in 2007[53] which recognizes the contribution of traditional medicine to a community's health and well-being was a step towards a this more flexible approach.[24] Health care facilities can also seek solutions for working with traditional healers and community education programs could encourage people to also consider seek treatment from clinics in addition to traditional methods.[44] As the framework of participatory development dictates, community members can be involved in the development and implementation phases so as improve the success of such programs by ensuring they are contextually relevant and act as advocates for the use of formal health care providers.[44] [54]

## Conclusion

Because the symptoms of malaria and early signs of pregnancy are often similar and overlapping, in addition to the commonness of malaria, knowledge of MiP risk is low in Madang. This, in addition to discomfort with the interventions, can lead to low levels of adherence to malaria prophylaxis or sleeping under mosquito nets. Furthermore, although health talks delivered in ANC waiting rooms provide women with important information about their general health and well-being, these messages and those concerning MiP are not well divided, which leads to misunderstandings and missed opportunities for prevention. Finally, concepts of illness as rooted in conflict and the mixing of traditional and biomedical prevention and treatment methods are key factors affecting the uptake of and adherence to MiP preventative interventions. Policy and health care facility practice must address the above issues in order to provide a comprehensive and successful MiP prevention program.
